# Decane-1,10-diaminium dinitrate

**DOI:** 10.1107/S1600536811042929

**Published:** 2011-10-22

**Authors:** Charmaine Arderne

**Affiliations:** a University of Johannesburg, Department of Chemistry, PO Box 524, Auckland Park, Johannesburg 2006, South Africa

## Abstract

The crystal structure of the title compound, C_10_H_26_N_2_^2+^·2NO_3_^−^, exhibits a back-to-back paired double-stacked packing arrangement culminating in an overall double zigzag pattern of the dications. Each pair of double-stacked dications is surrounded by a ring of ten nitrate anions. An intricate three-dimensional N---H...O and N---H...(O,O) hydrogen-bonding network exists in the crystal structure.

## Related literature

For related structural studies of *n*-alkyl-diammonium nitrate salts, see: van Blerk & Kruger (2009 ▶). The structures of many other related *n*-alkyl­diammonium salts are available in the Cambridge Structural Database, see: Allen (2002 ▶).
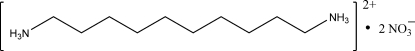


## Experimental

### 

#### Crystal data

C_10_H_26_N_2_^2+^·2NO_3_^−^
*M* *_r_* = 298.35
Monoclinic, 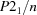

*a* = 5.4066 (5) Å
*b* = 20.3556 (16) Å
*c* = 14.5567 (11) Å
β = 93.853 (5)°
*V* = 1598.4 (2) Å^3^
*Z* = 4
Mo *K*α radiation
μ = 0.10 mm^−1^
*T* = 295 K
0.38 × 0.14 × 0.13 mm

#### Data collection

Bruker SMART CCD area-detector diffractometer
Absorption correction: multi-scan (*AXScale*; Bruker, 2008 ▶) *T*_min_ = 0.963, *T*_max_ = 0.987
36629 measured reflections
3857 independent reflections
1797 reflections with *I* > 2σ(*I*)
*R*_int_ = 0.086

#### Refinement

*R*[*F* ^2^ > 2σ(*F* ^2^)] = 0.055
*wR*(*F* ^2^) = 0.157
*S* = 1.02
3857 reflections
183 parameters
H-atom parameters constrained
Δρ_max_ = 0.16 e Å^−3^
Δρ_min_ = −0.19 e Å^−3^

### 

Data collection: *SMART-NT* (Bruker, 1999 ▶); cell refinement: *SAINT* (Bruker, 2008 ▶); data reduction: *SAINT*; program(s) used to solve structure: *SHELXS97* (Sheldrick, 2008 ▶); program(s) used to refine structure: *SHELXL97* (Sheldrick, 2008 ▶); molecular graphics: *OLEX2* (Dolomanov *et al.*, 2009 ▶ ) and *Mercury* (Macrae *et al.*, 2006 ▶); software used to prepare material for publication: *publCIF* (Westrip, 2010 ▶) and *PLATON* (Spek, 2009 ▶).

## Supplementary Material

Crystal structure: contains datablock(s) I, global. DOI: 10.1107/S1600536811042929/lr2031sup1.cif

Structure factors: contains datablock(s) I. DOI: 10.1107/S1600536811042929/lr2031Isup2.hkl

Supplementary material file. DOI: 10.1107/S1600536811042929/lr2031Isup3.mol

Supplementary material file. DOI: 10.1107/S1600536811042929/lr2031Isup4.cml

Additional supplementary materials:  crystallographic information; 3D view; checkCIF report

## Figures and Tables

**Table 1:** Hydrogen-bond geometry (Å, °)

*D*—H⋯*A*	*D*—H	H⋯*A*	*D*⋯*A*	*D*—H⋯*A*
N1—H1*C*⋯O2^i^	0.89	2.48	3.235 (3)	143
N1—H1*C*⋯O3^i^	0.89	2.05	2.892 (3)	158
N1—H1*E*⋯O4^i^	0.89	2.23	3.059 (3)	155
N1—H1*E*⋯O6^i^	0.89	2.37	2.969 (3)	125
N1—H1*D*⋯O4^ii^	0.89	2.00	2.867 (3)	163
N1—H1*D*⋯O5^ii^	0.89	2.51	3.255 (3)	141
N2—H2*C*⋯O1^iii^	0.89	2.36	3.063 (3)	136
N2—H2*C*⋯O3^iii^	0.89	2.12	2.983 (3)	163
N2—H2*D*⋯O1^iv^	0.89	1.99	2.849 (3)	163
N2—H2*E*⋯O6	0.89	1.97	2.838 (3)	166

Symmetry codes: (i) 
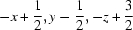
; (ii) 
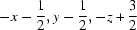
; (iii) 
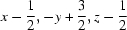
; (iv) 
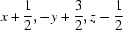
.

**Fig. 1.**
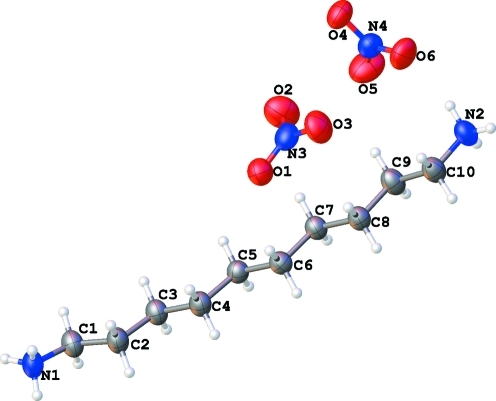
: Molecular structure of the title compound, with atomic numbering scheme and displacement ellipsoids drawn at the 50% probability level.

**Fig. 2.**
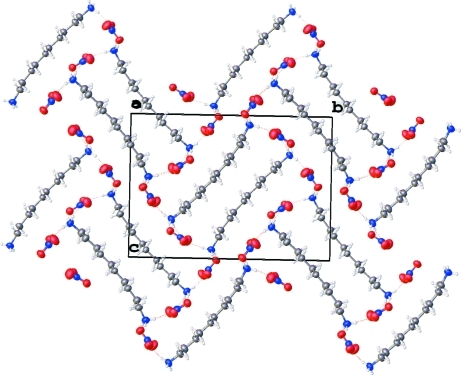
: Packing arrangement of the title compound viewed down the a axis. Selected hydrogen bonds are indicated by dashed lines.

**Fig. 3.**
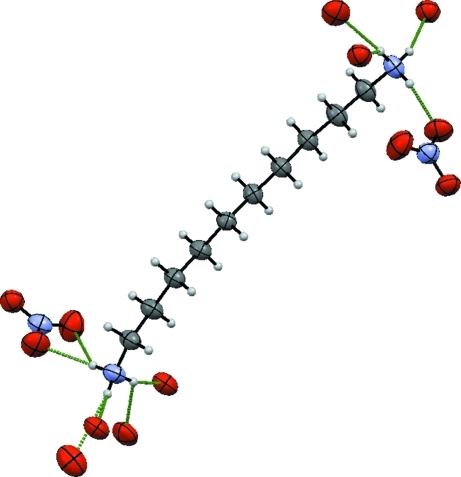
: Close-up view of the title compound clearly showing selected hydrogen-bonding interactions. Hydrogen bonds are indicated by green dashed lines.

## supplementary crystallographic information

### Comment

The crystal structure of the title compound, (I), adds to our current ongoing research of long-chained diammonium inorganic mineral acid salts. Colourless crystals of decane-1,10-diammonium dinitrate were obtained and analyzed by single-crystal X-ray diffraction techniques. This material forms part of our structural chemistry study of the inorganic mineral acid salts of the *n*-alkyldiamines. A search of the Cambridge Structural Database (Version 5.32, Allen, 2002) revealed that this compound had not previously been determined.

The asymmetric unit of compound (I) contains one diammonium dication and two nitrate anions with all atoms occupying general positions. The hydrocarbon chain is also fully extended with slight deviations from planarity chain as is evident from the torsion angles along the hydrocarbon chain (tabulated in Table 1). The molecular structure of (I) is shown in Figure 1.

Figure 2 illustrates the packing arrangement of the title compound (I) viewed down the *a* axis. The diammonium cations pack back-to-back, in pairs in a double zigzag pattern. Each dication pair is completely surrounded by a ring of nitrate anions. An extensive three-dimensional hydrogen-bonding network is also formed of N—H···O hydrogen bonds.

A close-up view of selected hydrogen bonding interactions can be viewed in Figure 3. The three-dimensional hydrogen bonding network is built and linked through hydrogen bonding interactions between the ammonium groups of the dication and the nitrate anions. Clear evidence of bifurcated hydrogen bonding interactions can also be seen in this illustration. The hydrogen bond distances and angles for the title compound (I) can be found in Table 2.

### Experimental

Compound (I) was prepared by adding decane-1,10-diamine (0.50 g, 2.90 mmol) to 55% nitric acid (2 ml, 42.5 mmol, Merck) in a sample vial. The mixture was then refluxed at 363 K for 2 h. The solution was cooled at 2 K h^-1^ to room temperature. Colourless crystals of decane-1,10-diammonium dinitrate were collected and a suitable single-crystal was selected for the X-ray diffraction study.

### Refinement

H atoms were geometrically positioned and refined in the riding-model approximation, with C—H = 0.97 Å, N—H = 0.89 Å, and *U*_iso_(H) = 1.2Ueq(C) or 1.5Ueq(N). For (I), the highest peak in the final difference map is 0.85Å from C6 and the deepest hole is 0.39Å from N2.

### Crystal data

**Table:**

C_10_H_26_N_2_^2+^·2NO_3_^−^	*F*(000) = 648
*M**_r_* = 298.35	*D*_x_ = 1.240 Mg m^−^^3^
Monoclinic, *P*2_1_/*n*	Mo *K*α radiation, λ = 0.71073 Å
Hall symbol: -P 2yn	Cell parameters from 1797 reflections
*a* = 5.4066 (5) Å	θ = 1.7–28.0°
*b* = 20.3556 (16) Å	µ = 0.10 mm^−^^1^
*c* = 14.5567 (11) Å	*T* = 295 K
β = 93.853 (5)°	Rectangular, colourless
*V* = 1598.4 (2) Å^3^	0.38 × 0.14 × 0.13 mm
*Z* = 4	

### Data collection

**Table:**

Bruker SMART CCD area-detector diffractometer	3857 independent reflections
Radiation source: fine-focus sealed tube	1797 reflections with *I* > 2σ(*I*)
graphite	*R*_int_ = 0.086
φ and ω scans	θ_max_ = 28.0°, θ_min_ = 1.7°
Absorption correction: multi-scan (AXScale; Bruker, 2008)	*h* = −7→7
*T*_min_ = 0.963, *T*_max_ = 0.987	*k* = −26→26
36629 measured reflections	*l* = −19→19

### Refinement

**Table:**

Refinement on *F*^2^	Primary atom site location: structure-invariant direct methods
Least-squares matrix: full	Secondary atom site location: difference Fourier map
*R*[*F*^2^ > 2σ(*F*^2^)] = 0.055	Hydrogen site location: inferred from neighbouring sites
*wR*(*F*^2^) = 0.157	H-atom parameters constrained
*S* = 1.02	*w* = 1/[σ^2^(*F*_o_^2^) + (0.0428*P*)^2^ + 1.010*P*] where *P* = (*F*_o_^2^ + 2*F*_c_^2^)/3
3857 reflections	(Δ/σ)_max_ = 0.001
183 parameters	Δρ_max_ = 0.16 e Å^−^^3^
0 restraints	Δρ_min_ = −0.19 e Å^−^^3^

### Special details

**Table:**

Geometry. All e.s.d.'s (except the e.s.d. in the dihedral angle between two l.s. planes) are estimated using the full covariance matrix. The cell e.s.d.'s are taken into account individually in the estimation of e.s.d.'s in distances, angles and torsion angles; correlations between e.s.d.'s in cell parameters are only used when they are defined by crystal symmetry. An approximate (isotropic) treatment of cell e.s.d.'s is used for estimating e.s.d.'s involving l.s. planes.
Refinement. Refinement of *F*^2^ against ALL reflections. The weighted *R*-factor *wR* and goodness of fit *S* are based on *F*^2^, conventional *R*-factors *R* are based on *F*, with *F* set to zero for negative *F*^2^. The threshold expression of *F*^2^ > σ(*F*^2^) is used only for calculating *R*-factors(gt) *etc*. and is not relevant to the choice of reflections for refinement. *R*-factors based on *F*^2^ are statistically about twice as large as those based on *F*, and *R*- factors based on ALL data will be even larger.

### Fractional atomic coordinates and isotropic or equivalent isotropic displacement parameters (Å^2^)

**Table:**

	*x*	*y*	*z*	*U*_iso_*/*U*_eq_	
C1	−0.3760 (5)	0.44497 (14)	0.84533 (18)	0.0520 (7)	
H1A	−0.4514	0.4872	0.8567	0.062*	
H1B	−0.4922	0.4196	0.8060	0.062*	
C2	−0.1406 (5)	0.45547 (13)	0.79673 (18)	0.0507 (7)	
H2A	−0.0804	0.4133	0.7766	0.061*	
H2B	−0.0150	0.4740	0.8399	0.061*	
C3	−0.1773 (5)	0.50071 (13)	0.71403 (17)	0.0481 (7)	
H3A	−0.2560	0.5409	0.7327	0.058*	
H3B	−0.2879	0.4797	0.6677	0.058*	
C4	0.0648 (5)	0.51777 (13)	0.67177 (18)	0.0482 (7)	
H4A	0.1809	0.5345	0.7198	0.058*	
H4B	0.1349	0.4779	0.6479	0.058*	
C5	0.0380 (5)	0.56815 (12)	0.59469 (17)	0.0429 (6)	
H5A	−0.0758	0.5511	0.5461	0.051*	
H5B	−0.0348	0.6078	0.6182	0.051*	
C6	0.2797 (5)	0.58597 (12)	0.55382 (17)	0.0440 (6)	
H6A	0.3980	0.5997	0.6031	0.053*	
H6B	0.3459	0.5471	0.5258	0.053*	
C7	0.2555 (5)	0.63977 (12)	0.48257 (17)	0.0453 (6)	
H7A	0.1883	0.6786	0.5106	0.054*	
H7B	0.1376	0.6259	0.4332	0.054*	
C8	0.4976 (5)	0.65833 (12)	0.44129 (18)	0.0451 (6)	
H8A	0.6183	0.6706	0.4907	0.054*	
H8B	0.5613	0.6202	0.4106	0.054*	
C9	0.4698 (5)	0.71431 (13)	0.3732 (2)	0.0534 (7)	
H9A	0.3583	0.7009	0.3217	0.064*	
H9B	0.3950	0.7514	0.4026	0.064*	
C10	0.7120 (5)	0.73582 (13)	0.33742 (19)	0.0521 (7)	
H10A	0.8249	0.7489	0.3888	0.063*	
H10B	0.7860	0.6991	0.3069	0.063*	
N1	−0.3260 (4)	0.40998 (11)	0.93400 (14)	0.0574 (6)	
H1C	−0.2968	0.3678	0.9231	0.086*	
H1D	−0.4570	0.4135	0.9676	0.086*	
H1E	−0.1942	0.4277	0.9645	0.086*	
N2	0.6789 (4)	0.79153 (10)	0.27172 (15)	0.0535 (6)	
H2C	0.5709	0.7802	0.2256	0.080*	
H2D	0.8238	0.8014	0.2496	0.080*	
H2E	0.6220	0.8263	0.3007	0.080*	
N3	0.6591 (5)	0.75622 (13)	0.64512 (16)	0.0567 (6)	
N4	0.3114 (5)	0.89735 (12)	0.42210 (17)	0.0569 (6)	
O1	0.6423 (4)	0.70426 (10)	0.68968 (14)	0.0644 (6)	
O2	0.4762 (4)	0.79050 (12)	0.62569 (18)	0.0889 (8)	
O3	0.8667 (4)	0.77376 (11)	0.62153 (16)	0.0784 (7)	
O4	0.2928 (4)	0.93497 (10)	0.49020 (14)	0.0663 (6)	
O5	0.1263 (5)	0.86953 (13)	0.38802 (17)	0.0916 (8)	
O6	0.5185 (4)	0.89060 (11)	0.39132 (14)	0.0746 (7)	

### Atomic displacement parameters (Å^2^)

**Table:**

	*U*^11^	*U*^22^	*U*^33^	*U*^12^	*U*^13^	*U*^23^
C1	0.0506 (17)	0.0611 (18)	0.0445 (15)	−0.0043 (13)	0.0048 (13)	0.0081 (13)
C2	0.0479 (17)	0.0566 (17)	0.0484 (15)	0.0036 (13)	0.0100 (13)	0.0087 (13)
C3	0.0459 (16)	0.0545 (16)	0.0442 (15)	0.0002 (13)	0.0044 (13)	0.0069 (13)
C4	0.0461 (16)	0.0532 (16)	0.0456 (15)	0.0036 (13)	0.0058 (13)	0.0066 (13)
C5	0.0422 (15)	0.0450 (14)	0.0414 (14)	0.0023 (12)	0.0027 (12)	0.0027 (11)
C6	0.0405 (15)	0.0442 (14)	0.0471 (15)	0.0007 (12)	0.0019 (12)	0.0043 (12)
C7	0.0430 (15)	0.0467 (15)	0.0463 (15)	0.0000 (12)	0.0048 (12)	0.0061 (12)
C8	0.0397 (15)	0.0482 (15)	0.0474 (15)	0.0021 (12)	0.0036 (12)	0.0070 (12)
C9	0.0429 (16)	0.0566 (17)	0.0611 (17)	0.0013 (13)	0.0065 (13)	0.0145 (14)
C10	0.0439 (16)	0.0526 (16)	0.0607 (17)	0.0031 (13)	0.0091 (14)	0.0117 (14)
N1	0.0617 (16)	0.0646 (15)	0.0476 (13)	0.0005 (12)	0.0160 (12)	0.0069 (12)
N2	0.0491 (14)	0.0523 (13)	0.0601 (14)	−0.0028 (11)	0.0109 (11)	0.0125 (11)
N3	0.0550 (17)	0.0659 (17)	0.0497 (14)	−0.0067 (14)	0.0067 (12)	−0.0110 (13)
N4	0.0588 (17)	0.0538 (15)	0.0593 (16)	0.0073 (13)	0.0115 (14)	−0.0001 (13)
O1	0.0645 (14)	0.0636 (13)	0.0666 (13)	−0.0052 (10)	0.0152 (11)	0.0005 (11)
O2	0.0615 (15)	0.0870 (17)	0.118 (2)	0.0084 (13)	0.0017 (14)	0.0138 (15)
O3	0.0573 (14)	0.0883 (16)	0.0919 (17)	−0.0133 (11)	0.0215 (12)	0.0101 (13)
O4	0.0644 (14)	0.0747 (14)	0.0612 (13)	0.0008 (11)	0.0147 (11)	−0.0154 (11)
O5	0.0677 (16)	0.0986 (18)	0.108 (2)	−0.0141 (14)	0.0001 (14)	−0.0358 (15)
O6	0.0610 (14)	0.0887 (16)	0.0768 (14)	0.0138 (12)	0.0241 (12)	−0.0092 (12)

### Geometric parameters (Å, °)

**Table:**

C1—N1	1.483 (3)	C8—C9	1.511 (3)
C1—C2	1.512 (3)	C8—H8A	0.9700
C1—H1A	0.9700	C8—H8B	0.9700
C1—H1B	0.9700	C9—C10	1.507 (3)
C2—C3	1.518 (3)	C9—H9A	0.9700
C2—H2A	0.9700	C9—H9B	0.9700
C2—H2B	0.9700	C10—N2	1.487 (3)
C3—C4	1.523 (3)	C10—H10A	0.9700
C3—H3A	0.9700	C10—H10B	0.9700
C3—H3B	0.9700	N1—H1C	0.8900
C4—C5	1.520 (3)	N1—H1D	0.8900
C4—H4A	0.9700	N1—H1E	0.8900
C4—H4B	0.9700	N2—H2C	0.8900
C5—C6	1.516 (3)	N2—H2D	0.8900
C5—H5A	0.9700	N2—H2E	0.8900
C5—H5B	0.9700	N3—O2	1.228 (3)
C6—C7	1.508 (3)	N3—O1	1.247 (3)
C6—H6A	0.9700	N3—O3	1.248 (3)
C6—H6B	0.9700	N4—O5	1.225 (3)
C7—C8	1.524 (3)	N4—O6	1.241 (3)
C7—H7A	0.9700	N4—O4	1.262 (3)
C7—H7B	0.9700		
			
N1—C1—C2	111.4 (2)	C6—C7—H7B	108.6
N1—C1—H1A	109.3	C8—C7—H7B	108.6
C2—C1—H1A	109.3	H7A—C7—H7B	107.6
N1—C1—H1B	109.3	C9—C8—C7	113.3 (2)
C2—C1—H1B	109.3	C9—C8—H8A	108.9
H1A—C1—H1B	108.0	C7—C8—H8A	108.9
C1—C2—C3	112.8 (2)	C9—C8—H8B	108.9
C1—C2—H2A	109.0	C7—C8—H8B	108.9
C3—C2—H2A	109.0	H8A—C8—H8B	107.7
C1—C2—H2B	109.0	C10—C9—C8	113.3 (2)
C3—C2—H2B	109.0	C10—C9—H9A	108.9
H2A—C2—H2B	107.8	C8—C9—H9A	108.9
C2—C3—C4	112.8 (2)	C10—C9—H9B	108.9
C2—C3—H3A	109.0	C8—C9—H9B	108.9
C4—C3—H3A	109.0	H9A—C9—H9B	107.7
C2—C3—H3B	109.0	N2—C10—C9	112.0 (2)
C4—C3—H3B	109.0	N2—C10—H10A	109.2
H3A—C3—H3B	107.8	C9—C10—H10A	109.2
C5—C4—C3	114.2 (2)	N2—C10—H10B	109.2
C5—C4—H4A	108.7	C9—C10—H10B	109.2
C3—C4—H4A	108.7	H10A—C10—H10B	107.9
C5—C4—H4B	108.7	C1—N1—H1C	109.5
C3—C4—H4B	108.7	C1—N1—H1D	109.5
H4A—C4—H4B	107.6	H1C—N1—H1D	109.5
C6—C5—C4	114.2 (2)	C1—N1—H1E	109.5
C6—C5—H5A	108.7	H1C—N1—H1E	109.5
C4—C5—H5A	108.7	H1D—N1—H1E	109.5
C6—C5—H5B	108.7	C10—N2—H2C	109.5
C4—C5—H5B	108.7	C10—N2—H2D	109.5
H5A—C5—H5B	107.6	H2C—N2—H2D	109.5
C7—C6—C5	114.0 (2)	C10—N2—H2E	109.5
C7—C6—H6A	108.8	H2C—N2—H2E	109.5
C5—C6—H6A	108.8	H2D—N2—H2E	109.5
C7—C6—H6B	108.8	O2—N3—O1	121.1 (3)
C5—C6—H6B	108.8	O2—N3—O3	120.0 (3)
H6A—C6—H6B	107.7	O1—N3—O3	118.9 (3)
C6—C7—C8	114.5 (2)	O5—N4—O6	122.3 (3)
C6—C7—H7A	108.6	O5—N4—O4	119.5 (2)
C8—C7—H7A	108.6	O6—N4—O4	118.2 (3)
			
N1—C1—C2—C3	−170.5 (2)	C5—C6—C7—C8	179.7 (2)
C1—C2—C3—C4	173.2 (2)	C6—C7—C8—C9	−177.5 (2)
C2—C3—C4—C5	−174.4 (2)	C7—C8—C9—C10	176.0 (2)
C3—C4—C5—C6	179.0 (2)	C8—C9—C10—N2	−179.2 (2)
C4—C5—C6—C7	−175.5 (2)		

### Hydrogen-bond geometry (Å, °)

**Table:**

*D*—H···*A*	*D*—H	H···*A*	*D*···*A*	*D*—H···*A*
N1—H1C···O2^i^	0.89	2.48	3.235 (3)	143
N1—H1C···O3^i^	0.89	2.05	2.892 (3)	158
N1—H1E···O4^i^	0.89	2.23	3.059 (3)	155
N1—H1E···O6^i^	0.89	2.37	2.969 (3)	125
N1—H1D···O4^ii^	0.89	2.00	2.867 (3)	163
N1—H1D···O5^ii^	0.89	2.51	3.255 (3)	141
N2—H2C···O1^iii^	0.89	2.36	3.063 (3)	136
N2—H2C···O3^iii^	0.89	2.12	2.983 (3)	163
N2—H2D···O1^iv^	0.89	1.99	2.849 (3)	163
N2—H2E···O6	0.89	1.97	2.838 (3)	166

Symmetry codes: (i) −*x*+1/2, *y*−1/2, −*z*+3/2; (ii) −*x*−1/2, *y*−1/2, −*z*+3/2; (iii) *x*−1/2, −*y*+3/2, *z*−1/2; (iv) *x*+1/2, −*y*+3/2, *z*−1/2.
